# Temperature Affects Biological Control Efficacy: A Microcosm Study of *Trichogramma achaeae*

**DOI:** 10.3390/insects12020095

**Published:** 2021-01-22

**Authors:** Long Chen, Annie Enkegaard, Jesper Givskov Sørensen

**Affiliations:** 1 Department of Agroecology, Aarhus University, 4200 Slagelse, Denmark; au620047@post.au.dk (L.C.); annie.enkegaard@agro.au.dk (A.E.); 2 Department of Biology, Aarhus University, 8000 Aarhus, Denmark

**Keywords:** fecundity, oviposition strategy, parasitisation, quality, thermal biology

## Abstract

**Simple Summary:**

The performance of biological control agents (BCAs) under field conditions is of importance to successfully suppress pests following release. However, the quality of BCAs is usually evaluated with laboratory measurements under controlled conditions, which has been shown unable to predict the performance in complex field conditions. In this study, we quantified the quality of the parasitoid *Trichogramma achaeae* in microcosms at four constant temperatures and evaluated its ability to locate and parasitise pest eggs. We also compared parasitisation efficiency with fecundity as determined under laboratory conditions. We found the biological control efficacy as determined in our microcosms was strongly regulated by temperature and was unlikely to be predicted by laboratory fecundity. These findings suggest that more complex assays, including behavioural responses, might be developed to demonstrate the field quality of BCAs.

**Abstract:**

Current quality control of mass-reared biological control agents (BCAs) is usually performed in the laboratory and often fails to include behavioural aspects of the BCAs. As a result, the use of efficacy measurements determined solely under laboratory conditions to predict field efficacy can be questioned. In this study, microcosms were designed to estimate biological control efficacy (realised parasitisation efficiency) of *Trichogramma achaeae* Nagaraja and Nagarkatti (Hymenoptera: Trichogrammatidae) parasitising *Ephestia kuehniella* Zeller (Lepidoptera: Pyralidae) eggs across the operational temperature range (15–30 °C). Temperature greatly affected the success of females in finding and parasitising *E. kuehniella* eggs, with parasitisation being reduced at 15 and 20 °C, as both the percentage of parasitised host eggs and the percentage of leaves per plant with parasitised host eggs decreased sharply compared with higher temperatures. Graphing previous data on laboratory fecundity against parasitisation efficiency shows that the laboratory-measured fecundity of *T. achaeae* was unlikely to predict field efficacy across temperatures. Results also showed that leaf side had no effect on the preference of *T. achaeae* in parasitising *E. kuehniella* eggs; however, *T. achaeae* preferred to lay their eggs on the top tier of plants. These findings suggest that more complex assays, which include behavioural responses, might be developed for optimised quality control of BCAs intended for field application.

## 1. Introduction

Mass rearing of biological control agents (BCAs) usually takes place in artificial environments, where constant climate conditions and factitious hosts or diets are applied to maximise productivity [1,2]. Accordingly, the likely adaptation of BCAs to rearing conditions might compromise performance under field conditions [3,4,5]. Therefore, quality control is essential for monitoring any quality change of BCAs [6]. Quality assays suggested by current quality control guidelines are usually carried out in the laboratory with easily measured life history parameters, such as fecundity, longevity, viability, and sex ratio, which are assumed to represent field control efficacy [3,7,8] but frequently fail to predict field performance [4,9]. Integrating measurements of behaviour, such as flight or locomotive activities, have been developed; however, studies linking these measures to field efficacy are few [10,11].

The potential discrepancy between laboratory and realised field performance is probably a consequence of the large variation in field conditions in comparison to standard laboratory conditions and the larger complexity of traits expressed in these field conditions (e.g., behavioural traits, which are usually not relevant in laboratory conditions). Specifically, laboratory measurements of quality parameters usually take place under controlled conditions at single constant temperatures [3], while field conditions are much more complex with climatic conditions fluctuating over time and space. For example, fecundity for parasitoids is often measured at favourable temperatures by giving animals immediate and infinite access to hosts [3,12], while, in nature, parasitoids are influenced by ambient temperature and the need to invest efforts in searching for the hosts which often results in low realised fecundity (e.g., [12,13]). Some studies applied fluctuating temperature environments to produce and evaluate the quality of BCAs and found that life history traits are generally affected by fluctuations [14,15,16,17]. Nevertheless, these life history traits are often measured under laboratory conditions, i.e., not incorporating behavioural aspects and with fecundity measured with ad libitum access to hosts, and they are difficult to convert to field performance. These issues question whether current measurements for quality can be reliably interpreted as field efficacy. Moreover, even when a behavioural component is considered thoroughly, e.g., in flight tests, the used assays are often quite simple, which likely fail to simulate a field-relevant behavioural component of the BCAs and the interactions between BCAs and pests [10,11,18].

Verifying relevant parameters and developing methods that are close to field conditions are required to comprehensively evaluate the potential efficacy of BCAs and maximise their quality [19]. Field assessment of realised control efficiency on relevant crops and crop pests would be ideal. This is, however, not always achieved as it can be both impractical and difficult to set up and score reliably. In this study, we used a simple microcosm approach with an artificial plant model, in an attempt to incorporate relevant behavioural aspects of the BCAs while keeping the experiment controlled and manageable. As a model BCA, we used the egg parasitoid *Trichogramma achaeae* Nagaraja and Nagarkatti (Hymenoptera: Trichogrammatidae). *Trichogramma* spp. are among those most successful biological control agents that have been widely and successfully used in biological control [20,21]. *T. achaeae* is a recently commercialised species reported as having a high efficacy in controlling the tomato leaf miner, *Tuta absoluta* Meyrick (Lepidoptera: Gelechiidae), a devastating pest in tomato production [22,23]. The aim of this study was to investigate the effect of different temperatures in an assay of performance that encompasses a more complex (microcosm) setting, allowing for behaviour to be included in the measure. We evaluated the assay relative to previously measured laboratory performance of the same species at the same temperatures [24], as a first step to link field biological control efficacy to simple laboratory parameters. We used an artificial plant model to test the search and parasitisation efficiency of *T. achaeae* against *Ephestia kuehniella* Zeller (Lepidoptera: Pyralidae) eggs at four constant temperatures (15, 20, 25, and 30 °C). We hypothesised that the realised parasitisation efficiency would follow a reaction norm with an optimal temperature around 25 °C. Furthermore, we asked if a standard measurement of fecundity in the laboratory would be predictive for the estimated biological control efficacy, which also includes aspects of the ability to locate hosts [25,26]. To account for the effects of distance, we investigated the effect of release point for the distribution of oviposition of *T. achaeae* in parasitising *E. kuehniella* eggs. We hypothesised that *T. achaeae* would have no preference and distribute oviposition across the whole plant, i.e., that parasitisation was not restricted by distance within a plant. We discuss the result in relation to previous work on simple life history parameters measured under the same temperature conditions as used here, and we discuss the implications of these results for predicting and optimising field application of *T. achaeae*. In summary, the main objectives of this study were (1) to investigate temperature effects of *T. achaeae* in parasitising host *E. kuehniella* eggs at four temperatures in a microcosm, (2) to determine if location of *E. kuehniella* eggs (on upper or lower surface of the leaf) influences *T. achaeae* parasitisation efficiency, and (3) to evaluate, using previously acquired data [24], if laboratory-measured fecundity can predict biological control efficacy in complex environments.

## 2. Materials and Methods

### 2.1. Experimental Animals

*T. achaeae* was ordered from Bioline AgroSciences Ltd., Essex, UK. At delivery, *T. achaeae* was in the pupal stage inside parasitised eggs glued to pieces of cardboard. The received animals were used directly without storage. Their factitious host *E. kuehniella* eggs (ultraviolet (UV) sterilised) were received in bulk in bottles from a commercial supplier (Koppert B.V., Berkel en Rodenrijs, The Netherlands). Eggs were stored at 4 °C for a maximum of 2 weeks until the next shipment arrived. Parasitoids were fed with 50% (*v*/*v*) organic honey solution as suggested by [27].

### 2.2. Laboratory Rearing

Parasitoids were reared continuously for a period of 2 months. Received *T. achaeae* cards were maintained in semi-transparent plastic bottles (approximately 300 mL) in a climate cabinet at 23 ± 1 °C, with a photoperiod of 12 h/12 h light/darkness. Bottles were positioned horizontally in a glass aquarium at the bottom of which was placed a petri dish (diameter: 9 cm) with saturated NaCl solution to maintain relative humidity (RH) 70% ± 10%. Initially, each bottle contained one card with approximately 2500 pupae. Upon emergence, newly emerged parasitoids (<24 h old) were provided with honey solution as food and with fresh *E. kuehniella* eggs glued on two cardboard pieces (1.3 × 6.3 cm/card, approximately 500 eggs/cm^2^) for oviposition for 24 h. Hereafter, the cards were transferred to new bottles and kept in the rearing aquarium as described above for adult emergence, after which the rearing cycle was repeated. The first parasitoids to be used for experimentation came from the second laboratory generation (i.e., after a full generation of rearing in the laboratory) to avoid systematic errors arising from the rearing conditions at the producer [28]. 

### 2.3. Microcosm Test of Biological Control Efficacy

To reduce the variations in size, morphology, and texture, artificial plants within plastic cylinders were used to test the efficiency of *T. achaeae* to find and parasitise *E. kuehniella* eggs (Figure S1, Supplementary Materials). Artificial plants were made by binding green paper card leaves (4.5 × 2.5 cm) to a bamboo stick (16.5 cm) with an intersection angle of 45°. In total, five leaves were attached in three tiers (top tier, middle tier, and bottom tier with one, two, and two leaves, respectively) at a distance of 4.3 cm between each tier. An egg card with six or seven *E. kuehniella* eggs was attached to each leaf, on either the upper or the lower side of the leaf tips. Hereafter, plant models were each inserted into a foam base (height: 3 cm) and individually set into transparent plastic cylinders (9 × 22.5 cm).

The experiment was conducted in climate cabinets at four constant temperatures of 15, 20, 25, and 30 ± 1 °C, with a photoperiod of 12 h/12 h light/darkness. Prior to experimentation, newly emerged parasitoids (less than 30 h old) were fed with honey and kept in bottles for an additional 3 h to ensure that all females were mated [29]. Females were then collected individually into small vials (4 mL) and randomly divided into four groups. Each group was transferred to a cabinet at one of the treatment temperatures for acclimation for 30 min before release into cylinders. The release took place directly in each cabinet to maintain the designated temperatures. For each treatment, a total of 32 replicates (16 for each leaf side) were set up. For each replicate, one single female parasitoid was introduced to the microcosm by knocking it down from the small vial to the top leaf of the plant. A fine honey drop was set on the top inner side as food in each cylinder. Afterwards, all cylinders were closed and maintained for 48 h at either of the above temperatures. Egg cards were collected and transferred to 25 °C, RH 70% ± 10%, with a photoperiod of 12 h/12 h light/darkness for development for 5 days. The realised parasitisation efficiency was then estimated as the percentage of parasitised eggs by counting the number of darkened (parasitised) relative to non-parasitised *E. kuehniella* eggs.

### 2.4. Data Analysis

All data were analysed using R (Version 3.6.2) [30]. The effect of temperature (linear or second-order polynomial) on realised microcosm parasitisation (percentage of leaves with parasitised eggs per plant and parasitisation efficiency) was analysed using linear models. Inspection of quantile–quantile (Q–Q) and residual plots was used to verify the assumptions for normality of residuals and homogeneity of variances. Selection of second-order polynomial versus linear models was based on Akaike information criterion (AIC) values. The distribution of parasitised eggs at different leaf sides or leaf tiers was analysed with Wilcoxon rank sum test (*p*-value adjustment method: Benjamini-Hochberg) for all replicates in which at least one *E. kuehniella* egg was parasitised.

## 3. Results

### 3.1. Realised Microcosm Parasitisation at Different Temperatures

**Figure 1:**
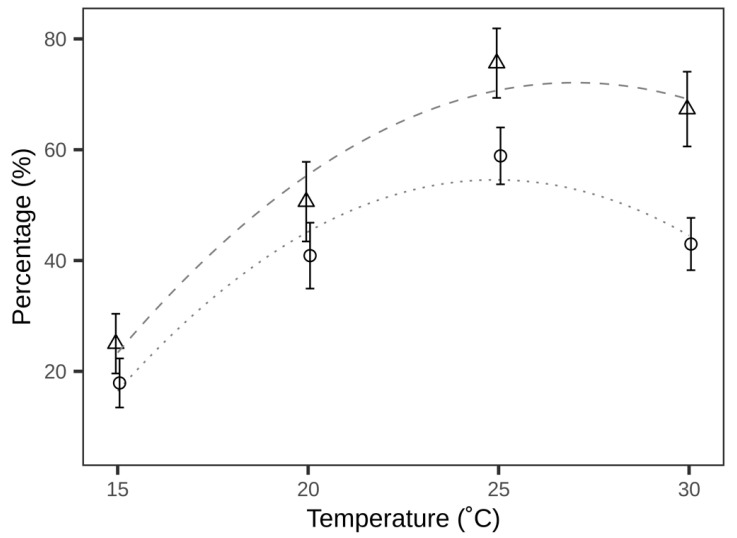
Percentage of leaves with parasitised eggs per plant (means ± standard error of the mean (SEM); triangles; dashed fitted line) and parasitisation efficiency (percentage of parasitised eggs) (means ± SEM; circles and dotted fitted line) at four constant temperatures. Fitted lines are predicted values according to second order polynomial linear models.

Temperature greatly affected the success of *T. achaeae* in finding and parasitising *E. kuehniella* eggs. The percentage of plants with one or more parasitised eggs was recorded as 56.25%, 68.75%, 90.62%, and 88.67%, for assays performed at 15, 20, 25, and 30 °C, respectively (Table 1). The average percentage of leaves with parasitised eggs per plant varied between 25.00% and 75.62% (Table 1). Model comparison showed a superior fit of the model including a second-order polynomial effect of temperature (AIC: 906.7) as compared to the linear model (AIC: 907.3). The selected model demonstrated a significant effect of temperature (ANOVA: F_(2, 123)_ = 17.59, *p* < 0.01), with the proportion of leaves with parasitised eggs declining at temperatures below or above 25 °C (Figure 1) and gave the following estimates (±standard error of the mean (SEM)): intercept, −174.46 ± 6.34; first order, 18.26 ± 5.81; second order, −0.34 ± 0.13. Likewise, the parasitisation efficiency (percentage of parasitised eggs) ranged from 17.9% to 58.9% (Table 1). Again, model comparison resulted in a better fit of the second-order polynomial temperature effect (AIC: 848.9) as compared to the model with a linear effect of temperature (AIC: 849.2). The selected model showed a significant effect of temperature (ANOVA: F_(2, 123)_ = 15.77, *p* < 0.01) with parasitisation efficiency declining at temperatures below or above 25 °C (Figure 1), and gave the following estimates (± SEM): intercept, −186.15 ± 49.57; first order, 19.33 ± 4.62; second order, −0.39 ± 0.10.

### 3.2. Distribution of Parasitised Eggs

No significant difference was found in either the percentage of leaves with parasitised eggs or the parasitisation efficiency between leaf sides (Wilcoxon rank sum test; Table 2 and Table S1, Supplementary Materials). However, the parasitisation efficiency at the top leaf tier of plants significantly exceeded that at other tiers at all testing temperatures except at 20 °C where no differences were found (Wilcoxon rank sum test; Table 2 and Table S1, Supplementary Materials). 

## 4. Discussion

**Figure 2:**
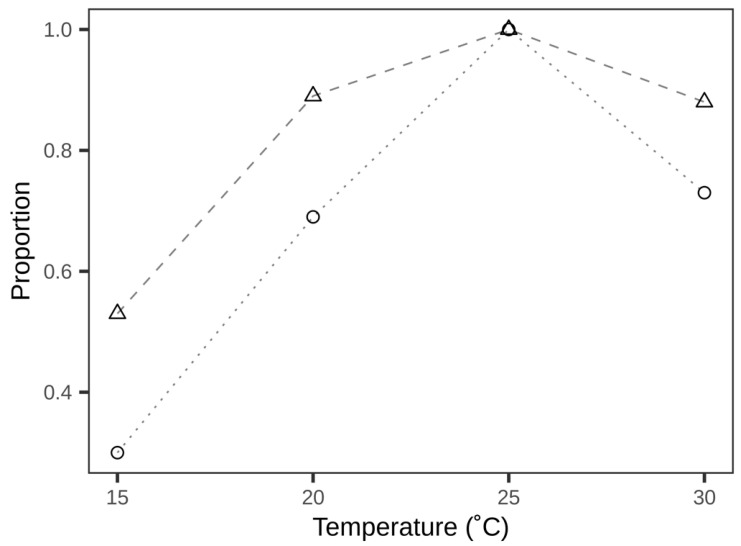
Comparison of relative laboratory fecundity (triangles and dashed line) from [24] and relative parasitisation efficiency (proportion of parasitised eggs) (circles and dotted lines) from this study. Relative values for each parameter were calculated by dividing absolute values at each temperature by the maximum (which occurred at 25 °C in both parameters).

Knowing how laboratory measurement of performance links to field efficacy may enhance quality control for BCAs and guide field applications. Previous studies on laboratory performance of *T. achaeae* demonstrated that several life history parameters (longevity, developmental time, sex ratio, body size, and fecundity) were strongly affected by temperature [24,31], and that fecundity best represented the overall laboratory performance [24]. In the microcosm study presented here, we show that the realised parasitisation ability in a microcosm was significantly influenced by temperature, similarly to that found for fecundity [24]. Assuming that the ability of parasitoids to find and parasitise host eggs in the microcosms could reflect biological control efficacy, we ask whether laboratory quality (fecundity) was able to predict “field performance” (microcosm parasitisation). By converting fecundity and parasitisation efficiency into relative values, a comparison of these two parameters was made (Figure 2). This comparison showed that, while both parameters changed qualitatively with temperature in a similar manner, parasitisation efficiency decreased more than fecundity as temperatures deviated from the optimal temperature of 25 °C. Considering the complexity of climate conditions in the field, this deviation in performance may be further amplified and, therefore, implies that laboratory-measured fecundity of *T. achaeae* is not a good predictor for field efficacy across temperatures, even if both parameters point to the same optimal temperature [12,32]. 

The discrepancy between relative fecundity and parasitisation efficiency exhibited in Figure 2 could be ascribed to the differences in measurements. Compared to laboratory measurement of fecundity, the method used in this study included more elements, e.g., searching behaviour, as both the percentage of leaves with parasitised eggs and the parasitisation efficiency (proportion of parasitised eggs) declined sharply below 20 °C (Table 1; Figure 1). Searching behaviour of insects comprises a series of actions, including locomotion (e.g., flight or walking) [33]. Flight activities have been reported in *Trichogramma sibericum* Sorkina to be affected by temperature, with decreasing temperature resulting in a reduced number of parasitoids initiating flight [25]. At the lowest temperature, flight was not observed in this study. Likewise, no flight was observed in *T. minutum* Riley when parasitoids were tested at 15 °C [34]. Moreover, walking activity was reported in *T. brassicae* Bezdenko to be negatively affected by decreasing temperature and greatly constrained when temperature was below 20 °C [26]. These studies verified that locomotive activities of *Trichogramma* parasitoids are strongly temperature-dependent. In this microcosm study, parasitoids were released from the top of cylinders and they had to either fly or walk to reach the distributed egg clusters. Low temperature might have affected these locomotive activities and led to the observed reduced parasitisation by *T. achaeae.* The results of the microcosm experiment also found a clear reduction in parasitisation ability at 30 °C. In comparison with our results, previous results on fecundity found no obvious difference at temperatures from 20 to 30 °C [24]. In view of the fact that the percentage of leaves with parasitised eggs remained the same from 25 to 30 °C, *T. achaeae* parasitised fewer eggs for each leaf at 30 °C. Consistent with a positive correlation between locomotion and temperature, an increased expenditure in energy for locomotion could divert resources and result in a reproductive rate [35,36]. This finding also indicates that high temperature at 30 °C would stress *T. achaeae* and subsequently decrease biological control efficacy; however, this negative effect of stress was concealed in laboratory measurements of fecundity, where fecundity was measured by giving parasitoids access to unlimited host eggs [24].

Variable temperatures have complex effects on insect performance. For instance, the development time of insects at constant temperatures is longer than the development time at fluctuating temperatures with the same mean temperature [37,38]. Previous studies found BCAs reared and tested under fluctuating temperatures to have greater longevity and higher fecundity compared with BCAs reared under equivalent constant temperatures [15,16]. Considering the benefits mentioned above, further studies on the consequences of fluctuating temperatures and how it relates to quality control, performance in microcosms as used in this study, and field performance are clearly warranted.

The preferred location of egg-laying of pests and their natural enemies affects biological control efficacy. For example, some pests lay eggs at the preferred position of leaves or other parts of plant [39,40], a behaviour which might affect the searching efficacy of natural enemies. A previous study reported that *T. achaeae* showed equal ability to locate *T. absoluta* eggs, regardless of which side the eggs were located [23]. This is in agreement with this study where leaf side had no influence on either percentage of leaves with parasitised eggs per plant or parasitisation efficiency across all temperatures. The current study suggests an advantage of *T. achaeae* in searching and parasitising *E. kuehniella* eggs on the apical plant parts—the same places preferred for egg-laying by their targeted pest, *T. absoluta* [41]. This finding would support the hypothesis that *T. achaeae* prefer to lay eggs on the top part of plant and suggest that releasing *T. achaeae* from lower part of plants could be more efficient to control pests for a whole plant. However, the result should be cautiously interpreted as *T. achaeae* was released at the top of the microcosm. Thus, while the parasitoids in most cases were able to reach and parasitise lower parts of the plant, we cannot dismiss the possibility of a distance effect.

Our study showed that temperature strongly affected the success of *T. achaeae* in finding and parasitising *E. kuehniella* eggs. The temperature-dependent parasitisation efficiency makes it difficult to predict field efficacy from laboratory-measured quality, indicating a need for updating the current quality control methods. Results also showed that leaf side had no effect on the preference of *T. achaeae* in parasitising *E. kuehniella* eggs; however, *T. achaeae* parasitised more eggs located on the top tier of plants. Although the designed microcosm differs from field conditions, it presents a potential method for differentiating temperature effects on searching behaviour and oviposition strategies that could be used in the quality control of mass-reared *Trichogramma spp*. Accordingly, considering the complex climate conditions in the field, and interactions of plant, pest, and natural enemies, this may have strong implications for field application and warrant further study on linking laboratory parameters to field success.

## Figures and Tables

**Table 1:** *Trichogramma achaeae* parasitising *Ephestia kuehniella* eggs: number (percentage) of plants with parasitised eggs, mean (±standard error of the mean (SEM)) percentage of leaves with parasitised eggs per plant and parasitisation efficiency (percentage of parasitised eggs) at four constant temperatures.

Temperature (°C)	Number of Plants Used	Number (and %) of Plants with Parasitised Eggs	Leaves with Parasitised Eggs (%)	Parasitisation Efficiency (%)
15	32	18 (56.25)	25.00 ± 5.39	17.90 ± 4.43
20	32	22 (68.75)	50.62 ± 7.18	40.88 ± 5.95
25	32	29 (90.62)	75.62 ± 6.27	58.89 ± 5.13
30	30	26 (88.67)	67.33 ± 6.75	42.98 ± 4.72

**Table 2:** Location of *Ephestia kuehniella* eggs parasitised by *Trichogramma achaeae*: mean (± SEM) percentage of leaves with parasitised egg per plant and parasitisation efficiency (percentage of parasitised eggs) by side of leaf (upper or lower) or leaf placement on plants at four constant temperatures. Different letters indicate significantly differences between Upper and Lower leaf side and/or between Top, Middle and Bottom leaf tier within temperatures, respectively (*p* < 0.05, Wilcoxon rank sum test).

Temperature (°C)	Number of Plants (Per Leaf Side)	Number of Plants with Parasitised Eggs		Leaves with Parasitised Eggs (%)		Parasitisation Efficiency (%)				
	*n*	Upper	Lower	Upper	Lower	Upper	Lower	Top Tier	Middle Tier	Bottom Tier
15	16	10	8	42.00 ± 9.21 a	47.50 ± 9.64 a	32.46 ± 9.67 a	31.03 ± 7.41 a	51.40 ± 9.23 a	35.45 ± 7.84 ab	19.82 ± 6.83 b
20	16	12	10	70.00 ± 7.18 a	78.00 ± 8.67 a	54.82 ± 5.79 a	65.04 ± 8.01 a	70.67 ± 8.34 a	64.39 ± 5.46 a	48.16 ± 7.94 a
25	16	14	15	80.00 ± 6.95 a	86.67 ± 7.22 a	61.50 ± 5.39 a	68.22 ± 6.54 a	81.53 ± 3.84 a	66.40 ± 5.05 b	55.42 ± 6.64 b
30	15	13	13	70.77 ± 8.36 a	84.62 ± 6.47 a	47.11 ± 6.08 a	52.06 ± 5.63 a	69.12 ± 4.92 a	44.81 ± 5.72 b	43.68 ± 4.70 b

## Data Availability

The data presented in this study are available in the supplementary material of this paper.

## Supplementary Materials

The following are available online at https://www.mdpi.com/2075-4450/12/2/95/s1. Figure S1. The microcosm set-up for testing realised parasitisation efficiency of *Trichogramma achaeae*; Table S1. *p*-Values from Wilcoxon rank sum test comparing upper vs. lower leaf side for percentage of leaves with parasitised eggs and parasitisation efficiency (percentage of parasitised eggs) and comparison of leaf tiers for parasitisation efficiency within each treatment (constant rearing temperature); Excel sheet of experimental data.
