# Supplementary material for: Temperature Affects Biological Control Efficacy: A Microcosm Study of Trichogramma achaeae
Source: Insects. 2021 Jan 22;12(2):95. doi: 10.3390/insects12020095 (PMC7912409; doi:10.3390/insects12020095)
Supplement: Supplementary file 1 [file insects-12-00095-s001.pdf]

Article

Temperature Affects Biological Control Efficacy: A Microcosm Study of *Trichogramma achaeae*

Long Chen [1,2], Annie Enkegaard [1] and Jesper Givskov Sørensen [2,*]

[1] Department of Agroecology, Aarhus University, 4200 Slagelse, Denmark; annie.enkegaard@agro.au.dk (A.E.)
[2] Department of Biology, Aarhus University, 8000 Aarhus, Denmark; au620047@post.au.dk (L.C.); jesper.soerensen@bio.au.dk (J.G.S.)
* Correspondence: jesper.soerensen@bio.au.dk; Tel.: +45-87156532

Supplementary Materials

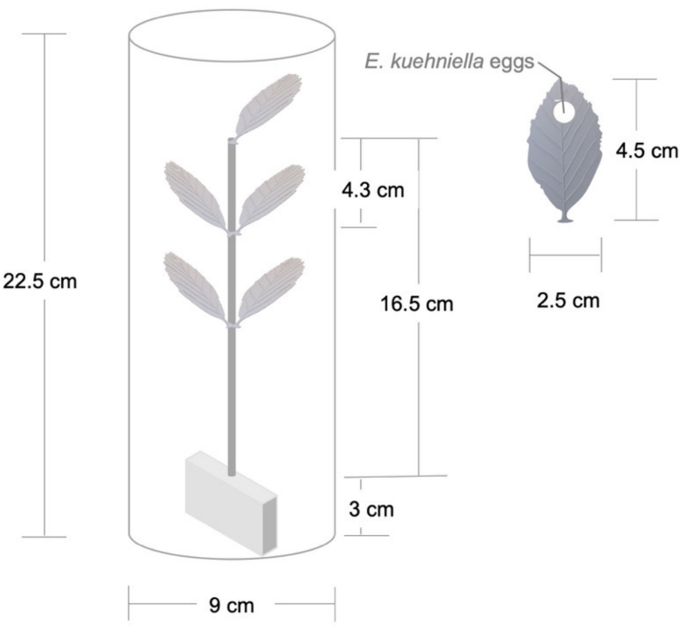

Figure S1. The microcosm set-up for testing realised parasitisation efficiency of *Trichogramma achaeae*. An artificial plant with 5 paper leaves was set into a cylinder, and for each cylinder, a single female was released for parasitisation for 48 h.

Table S1. P values from Wilcoxon rank sum test comparing upper vs. lower leaf side for percentage of leaves with parasitised eggs and parasitisation efficiency (percentage of parasitised eggs) and comparison of leaf tiers for parasitisation efficiency within each treatment (constant rearing temperature). *: $p < 0.05$.

Temp (°C)	Leaf sides		Leaf tiers		
			Parasitisation efficiency		
	Percentage of leaves with parasitised egg	Parasitisation efficiency	Top vs middle	Top vs bottom	Middle vs bottom
15	0.5986	0.8938	0.305	0.033*	0.188
20	0.3262	0.1759	0.25	0.15	0.25
25	0.3657	0.1688	0.0068*	0.0068*	0.5515
30	0.2153	0.6628	0.0052*	0.0004*	0.9634
